# Hidradenitis Suppurativa and IL-17 Inhibitors: A Focus on Trials and Real-Life Data

**DOI:** 10.3390/medicina62050952

**Published:** 2026-05-13

**Authors:** Fabrizio Martora, Nello Tommasino, Valeria Esposito, Claudio Brescia, Matteo Megna

**Affiliations:** Section of Dermatology, Department of Clinical Medicine and Surgery, University of Naples Federico II, Via Pansini 5, 80131 Naples, Italy; nello.tommasino@libero.it (N.T.); espositovaleria27@gmail.com (V.E.); dott.claudiobrescia@gmail.com (C.B.); mat24@libero.it (M.M.)

**Keywords:** hidradenitis suppurativa, oral therapy, IL-17, secukinumab, bimekizumab

## Abstract

Hidradenitis suppurativa (HS) is a chronic, relapsing inflammatory skin disease associated with significant physical and psychosocial burden. Increasing evidence identifies the interleukin (IL)-17 pathway as a central driver of HS pathogenesis. Elevated levels of IL-17 family cytokines, particularly IL-17A and IL-17F, have been consistently demonstrated in lesional skin, where they contribute to keratinocyte activation, neutrophil recruitment, and amplification of proinflammatory cascades. This review provides a comprehensive overview of the pathogenic role of IL-17 in HS and summarizes current evidence on IL-17-targeted therapies from clinical trials and real-world studies. Secukinumab, a selective IL-17A inhibitor, has shown significant efficacy in phase III trials, with clinically meaningful improvements in Hidradenitis Suppurativa Clinical Response (HiSCR), pain, and quality of life, sustained over long-term follow-up. Bimekizumab, a dual inhibitor of IL-17A and IL-17F, has demonstrated robust and consistent efficacy across multiple endpoints, including higher response rates and greater improvements in patient-reported outcomes, supporting the relevance of dual cytokine inhibition. Despite these advances, treatment responses remain heterogeneous, reflecting the multifactorial nature of HS. Further research is needed to identify predictive biomarkers and optimize therapeutic strategies.

## 1. Introduction

Hidradenitis suppurativa (HS) is a chronic inflammatory skin disease characterized by persistent or recurring painful inflammatory nodules, abscesses and draining tunnels in the apocrine gland-rich areas of the body such as axillary, inguinal, genital or inframammary regions [1,2].

This disorder typically starts in the second or third decade of life. Data on the prevalence of HS are controversial, ranging from 0.05% to 4.1%. Furthermore, European studies estimate the prevalence at 1%, ten times higher than in the United States [3]. Other American studies report prevalence rates between 0.4% and 0.58% [4,5]. However, a recent meta-analysis including 22,743 participants and identifying 247 patients with HS across 23 countries over 6 continents estimated a global prevalence of HS between 0.67% and 1.46% [6].

In the European and North American populations, the male-to-female ratio is 1:3, while in South Korea it is reversed at 2:1 [3].

Due to its chronic nature and frequently occurring relapses, HS has a significant impact on quality of life of the affecting both personal and professional aspects. It is also associated with several comorbidities, including metabolic syndrome, type 2 diabetes, arteriosclerosis, depression, anxiety, and inflammatory bowel disease [7].

Diagnosis and staging of HS are primarily clinical, although high frequency ultrasound can support more accurate severity assessment [8]. HS can be broadly classified into inflammatory and predominantly non-inflammatory forms. Disease severity in the inflammatory form is assessed using the International Hidradenitis Suppurativa Severity Score System (IHS4), while the non-inflammatory form is often managed surgically according to Hurley staging, which classifies disease severity into three stages based on the presence of abscesses, sinus tracts, and scarring [9,10].

Management of HS remains challenging due to its chronic relapsing nature and the still incompletely understood pathophysiology. In addition, diagnostic delays are common and often lead to disease progression and extensive scarring, further complicating treatment.

The etiology of HS is multifactorial, involving both genetic and environmental factors. Among lifestyle factors, smoking and obesity play a key role in disease onset and progression. Approximately one-third of patients show a genetic predisposition, often involving mutations affecting the γ-secretase complex or keratinocyte dysfunction, leading to a pro-inflammatory and hyperproliferative phenotype [11].

The primary pathogenic event in HS is follicular occlusion, leading to dilation and subsequent rupture of the hair follicle. This results in the release of keratin and bacteria into the dermis, triggering an intense inflammatory response characterized by neutrophil and lymphocyte infiltration, abscess formation, and destruction of the pilosebaceous unit [12].

Both innate and adaptive immune dysregulation contribute to HS pathogenesis. Increased levels of pro-inflammatory cytokines (including IL-1β, TNF-α, IL-23, and IL-17) as well as anti-inflammatory cytokines (such as IL-10) have been detected in lesional and perilesional skin [13].

Th1 and Th17 cells play a central role in HS inflammation. TNF-α acts as an upstream mediator promoting T-cell differentiation into Th1 and Th17 subsets, which produce interferon (IFN)-γ and IL-17, respectively [14].

Targeted immunomodulatory therapies directed against these cytokines have emerged as effective treatment options. In particular, the IL-17 pathway has been identified as a key driver in HS pathogenesis, leading to the development and approval of IL-17 inhibitors for HS treatment. The aim of this review is to summarize the pathophysiological role of IL-17 in HS and to critically evaluate therapeutic strategies targeting this pathway.

## 2. IL-17

In recent years, increased levels of IL-17 and IL-23 (which promotes IL-17 production) have been observed in HS lesions, supporting a key role for Th17 cells in disease pathogenesis. Indeed, lesional HS skin shows significant immune cell infiltration, including a high number of Th17 cells, compared with non-lesional skin [15].

IL-17 signaling promotes the expression of antimicrobial peptides (S100A8, S100A9, and human β-defensin-2), as well as chemokines such as CXCL1 and IL-8, all of which are upregulated in HS lesions and contribute to inflammation [16].

Keratinocytes represent a major target of IL-17. Through activation of the NLRP3 inflammasome and caspase-1, IL-17 stimulates keratinocytes to produce IL-1β, chemokines, and antimicrobial peptides. In addition, IL-36 cytokines, which belong to the IL-1 family, further amplify Th1 and Th17 responses, suggesting the presence of a self-perpetuating inflammatory loop involving IL-1 and IL-17 pathways [17]. Six members of the IL-17 family have been identified (IL-17A–F), among which IL-17A, IL-17C, and IL-17F appear to play the most relevant roles in HS [18]. IL-17A, a hallmark cytokine of Th17 cells, is significantly overexpressed in HS lesions. For example, Schlapbach et al. reported a 30-fold increase in IL-17A gene expression in lesional skin compared with healthy controls [14], while Kelly et al. demonstrated a 149-fold increase in IL-17A mRNA expression [19,20].

Both IL-17A and IL-17F are elevated in HS lesions, particularly in the dermis and in association with draining tunnels [21]. Notably, IL-17F appears to be more abundantly expressed than IL-17A, especially in chronic lesions such as nodules, abscesses, and sinus tracts [22].

Moreover, circulating levels of IL-17F have been found to be higher than IL-17A in patients with HS, further supporting its potential role in sustaining chronic inflammation [21]. Compared with psoriasis, HS-derived Th17 cells produce significantly higher levels of IL-17F [23].

In summary, analyses of human clinical samples and in vitro preclinical experiments demonstrate that both IL-17A and IL-17F are upregulated in HS lesional skin compared with non-lesional and healthy control skin, with expression detected primarily within inflammatory infiltrates and lesional tissue compartments. Importantly, IL-17F appears to be expressed at higher levels than IL-17A in HS lesions. Despite sharing the same receptor complex (IL-17RA/IL-17RC) and overlapping downstream signalling pathways, IL-17F is quantitatively more abundant in lesional tissue, suggesting that it may play a particularly relevant role in sustaining chronic inflammation [24].

IL-17C also contributes to HS pathogenesis. It is produced by keratinocytes following stimulation by IL-17A/F, TNF, or bacterial signals and promotes the release of IL-1β, IL-8, CXCL1, and IL-36γ, further amplifying Th17 responses and creating a self-sustaining inflammatory loop [25].

Dermal tunnels, a hallmark of chronic HS, show increased expression of IL-17C, IL-1A, IL-1B, and IL-6, contributing to disease persistence. These cytokines interact with fibroblast-derived mediators to sustain Th17-driven inflammation [23].

Moreover, it has been found that IL-26, a cytokine associated with Th17 cells, is increased in the plasma and skin of patients with HS. This has direct antimicrobial activity, thereby contributing to a deficient antimicrobial response in HS [26].

These mechanistic insights provide a strong rationale for targeting the IL-17 pathway in HS. Selective inhibition of IL-17A (e.g., secukinumab) reduces inflammatory activity, while dual inhibition of IL-17A and IL-17F (e.g., bimekizumab) may provide broader suppression of the inflammatory cascade. However, the complexity of HS pathogenesis likely contributes to the variability in treatment response observed in clinical practice. The identification of reliable biomarkers and predictors of response remains a major unmet need in HS management. To date, no validated biomarkers are available to guide therapeutic selection, limiting the implementation of personalized treatment strategies [9].

## 3. Materials and Methods

This review was conducted following a structured approach inspired by the PRISMA guidelines to systematically evaluate the efficacy and safety of IL-17 inhibitors in hidradenitis suppurativa (HS).

A literature search was performed in the PubMed database for articles published up to January 2026, using the keywords “hidradenitis suppurativa”, “IL-17”, “secukinumab”, “bimekizumab”, “ixekizumab”, “brodalumab”, “clinical trial”, and “real-world study”. Reference lists of relevant articles were also screened.

Studies were included if they involved patients with HS treated with IL-17 inhibitors and reported clinical efficacy or safety outcomes (e.g., HiSCR, IHS4, pain, quality of life). Randomized controlled trials and observational studies were considered, while non-English articles and studies without primary data were excluded.

Data on study design, patient characteristics, treatment regimens, and outcomes were extracted and qualitatively synthesized due to heterogeneity across studies. The primary outcome was HiSCR, with secondary outcomes including IHS4, pain reduction, and quality-of-life measures.

## 4. Secukinumab Trials

In 2019, Prussick et al. conducted a 24-week pilot study including 9 patients with Hurley stage II–III HS who had failed at least one course of antibiotic therapy [27]. The primary endpoint was achievement of Hidradenitis Suppurativa Clinical Response (HiSCR50), defined as a ≥50% reduction in inflammatory lesion count with no increase in abscesses or draining fistulas compared with baseline [28]. At week 24, 78% of patients achieved HiSCR50, with no serious adverse events (AEs) reported [7]. Similarly, Casseres et al. reported a HiSCR50 rate of 70% at week 24 in a cohort of 20 patients, again without serious AEs [29]. Subsequently, the phase III randomized, double-blind, placebo-controlled trials SUNSHINE and SUNRISE evaluated secukinumab in larger patient populations. A total of 541 and 543 patients were enrolled, respectively, across 219 sites in 40 countries [30]. Eligible patients were adults with moderate-to-severe HS, defined by the presence of at least five inflammatory lesions in at least two anatomical areas. Patients with more than 20 fistulas or receiving prohibited therapies were excluded [30]. Participants were randomized (1:1:1) to receive secukinumab every 2 weeks (SECQ2W), every 4 weeks (SECQ4W), or placebo. The primary endpoint was HiSCR50 at week 16. In SUNSHINE, HiSCR50 was achieved by 45% of patients in the SECQ2W group and 42% in the SECQ4W group, compared with 34% in the placebo group (odds ratio [OR] 1.8 for SECQ2W) [30]. In SUNRISE, response rates were 42% (OR 1.6) for SECQ2W and 46% (OR 1.9) for SECQ4W, versus 31% for placebo [30]. Clinical responses were sustained through week 52. In SUNSHINE, HiSCR50 was achieved by 56.4% (SECQ2W) and 56.3% (SECQ4W), while in SUNRISE the corresponding rates were 65% and 62.2% [30]. A pooled analysis by Zouboulis et al. [31] stratified 1084 patients into biologic-naïve (n = 829) and biologic-experienced (n = 255) subgroups. HiSCR50 response rates were consistently higher with secukinumab than placebo in both groups. Among biologic-naïve patients, response rates were 45.6% (SECQ2W), 45.4% (SECQ4W), and 34.2% (placebo), while among biologic-experienced patients they were 37.0%, 38.8%, and 27.3%, respectively [31]. Responses were maintained up to week 52. Passera et al. [32] performed an automated analysis of the data from the two trials, dividing patients into three clusters. Cluster 1 (54.1% of patients) included patients with milder HS and more women (65.4%); Cluster 2 had 58.5% of patients from the Middle East, Africa, and Pacific Asia; Cluster 3 was characterized by more severe HS, the highest percentage of bio-experienced patients (45.9%), and previous surgeries (47.5%). In all clusters at week 16, SECQ2W and SECQ4W proved to be more effective than placebo, with results maintained until week 52 [32]. A post hoc analysis of the SUNSHINE and SUNRISE trials evaluated the International Hidradenitis Suppurativa Severity Score System (IHS4) to compare the efficacy of secukinumab in study patients. At week 16, the mean reduction in IHS4 was −10.80 with SECQ2W, −9.46 with SECQ4W, and −4.92 with placebo. A 55% reduction in IHS4 (IHS4-55) was achieved by 43.4%, 39.5%, and 31.5% of the respective groups, with further improvements at week 52 [33]. At week 16, the percentage of patients with mild HS, who were not present at baseline as they all had moderate-to-severe HS, increased to 25.9% with SECQ2W and 24.0% with SECQ4W, higher than 16.4% in the placebo group [33]. Among the secondary endpoints, reduction in skin pain was assessed using the Numeric Rating Scale (NRS). Ingram et al. observed that at week 16 there was a greater reduction in the mean NRS with SECQ2W (−1.35) and SECQ4W (−1.05) compared to placebo (−0.47) [34]. Wei et al. also evaluated the NRS30, which is the reduction of ≥30% and ≥2 points in skin pain. About 41–45% of patients treated with secukinumab achieved an NRS30 at week 16, compared with around 28% in the placebo group, demonstrating a significantly greater reduction in pain among secukinumab-treated patients [35]. Achieving NRS30 at week 16 also predicted a higher likelihood of improved quality of life. The response was maintained through week 52 with a trend towards further improvement, indicating sustained pain relief over time [35]. Kimball et al. evaluated the time to loss of response (LOR) up to week 104 in the SUNSHINE and SUNRISE extension trials. SECQ2W and SECQ4W patients who reached HiSCR50 at week 52 were randomized 2:1 to continue secukinumab or receive placebo. The median time to LOR was around 283 days for the Q2W continuation arm and 365 days for the Q4W continuation arm, compared with 239 days and 171 days for their respective placebo arms [36]. At the time of LOR, a considerable proportion of patients still maintained HiSCR50: 44% in the Q2W continuation arm, 58% in the corresponding placebo arm, 40% in the Q4W continuation arm, and 34% in the Q4W placebo arm [36]. Alavi et al. evaluated the pharmacokinetics of secukinumab and serum C-reactive protein (CRP) concentrations based on data from these trials. The results showed that trough serum concentrations of secukinumab at weeks 16, 24 and 52 were approximately twice as high in the Q2W group compared to the Q4W group. Despite this difference, at week 16 the overlap in exposure between dosing regimens was substantial, and only a modest numerical (~3%) increase in clinical response (HiSCR50) was estimated for the Q2W regimen. By week 52, the HiSCR50 responses had plateaued [37]. Changes in CRP were also documented: in the Q2W arm hsCRP decreased from approximately 18.6 mg/L to 12.8 mg/L, and in the Q4W arm from approximately 15.9 mg/L to 11.5 mg/L by week 16; these reductions were maintained through week 52 whereas the placebo arm remained essentially unchanged. It was further observed that higher body weight, greater disease severity and elevated baseline hsCRP were each associated with lower secukinumab serum concentrations [37]. With regard to safety, no unexpected AEs were reported, and the most common was headache in both trials. In SUNSHINE, it was observed in 8% of the placebo group, 9% of SECQ2W, and 11% of SECQ4W [30]. In SUNRISE it occurred in 8%, 12% e 9% of the respective groups [30]. In the analysis by Zouboulis et al. of the biologic-naïve and biologic-experienced subgroups there were no significant variations in safety between them [31]. Even in the extended trials at week 104 the safety profile of secukinumab through week 104 was consistent with the core trials, and no new safety signals were identified [36].

## 5. Secukinumab Real Life Studies

The efficacy and safety of secukinumab against HS has also been evaluated in several real-world studies. Reguiaï et al. observed that out of 20 patients after 16 weeks of treatment, 75% achieved HiSCR50 and the HS-Physician Global Assessment (PGA) decreased from 4 to 2 [38]. No flare-ups were observed after a mean follow-up of 14 (3–36) months, but two patients discontinued treatment due to the onset of Crohn’s disease [38]. In the results published by Fernandez-Crehuet et al. from a retrospective multicenter study of 47 patients treated with secukinumab, HiSCR50 was achieved in 48.9% of cases [39]. The authors also used multivariate analysis to highlight that this outcome was more likely to be achieved by women and by those with a lower disease burden in terms of both extent and severity [39]. AEs were observed in 3 patients (6.8%), of whom 2 discontinued treatment due to the onset of paradoxical psoriasis and joint pain. Only in one case was secukinumab discontinued due to worsening of HS, while in 12.8% of cases it was discontinued due to ineffectiveness [39]. In a multi-center retrospective study, Ribero et al. evaluated 31 patients treated with secukinumab, all of whom had either failed previous anti-TNF-alpha therapies or had contraindications to them. At week 28, 41% of the patients achieved the HiSCR50. The treatment was reported to be safe, with no AEs recorded [40]. The same endpoint was achieved after 16 weeks by 57.1% of patients enrolled in the study by Martora et al. These patients treated with secukinumab had already failed adalimumab for HS. Follow-up in this single-center study continued until week 52, when the endpoint was achieved by 71.4% of patients. The secondary endpoints IHS4, DLQI, and VAS pain scale also improved at weeks 16 and 52 compared to baseline [41]. Haselburger et al. enrolled 67 patients with HS in a multicenter study. At week 24, 41.8% of patients achieved HiSCR50, while 44.8% achieved IHS4-55. Regarding safety, 10.5% of patients experienced AEs, with 3 patients discontinuing secukinumab [42]. Roccuzzo et al. conducted a long-term analysis of the efficacy of secukinumab in HS in 24 patients. HiSCR50 and IHS4-55 were achieved by 25% and 8.3% of patients after 12 weeks and 56.5% and 54.2% after 24 weeks, respectively. At 48 weeks, however, both values decreased to 28.5% [43]. A Spanish single-center study of 23 patients with HS evaluated HiSCR50 at weeks 16, 24, and 52, which was achieved by 73.9%, 71.4%, and 83.3% of patients, respectively. The mean IHS4 also decreased from 11.4 ± 9.3 at baseline to 5.8 ± 7.1, 5.6 ± 7.8, and 4.73 ± 7.9 in the respective periods [44]. Only one patient discontinued due to AEs (arthritis), while two discontinued due to primary ineffectiveness and another due to secondary failure at week 36 [44]. Melgosa Ramos et al. then evaluated 14 patients who had previously failed conventional or biological therapy for HS. After 12 weeks of secukinumab, HiSCR50 was achieved by 85% of patients. At week 36, this figure fell to 71.4% due to secondary failure in 2 patients. No relevant AEs were observed [45]. In the retrospective, single-center observational study by Tran et al., 46 patients who had previously failed anti-TNF-α therapy were recruited. The primary outcome was the observation of a significant response after 16 weeks of secukinumab, defined as improvement in existing lesions, no new lesions, or no extension. This objective was achieved by 43.5% of patients [46]. Patients with Hurley stage III disease who received both surgery and secukinumab showed a markedly improved response rate, according to comparative analyses [46]. In a multicenter study conducted in 2025, Erbağcı et al. recruited 31 patients with HS. After 12 weeks of treatment with secukinumab, 64.5% of patients achieved HiSCR50, while in 29% of patients the drug was discontinued due to primary failure. Only 1 patient (3.2%) discontinued due to secondary ineffectiveness and another due to paradoxical pyoderma gangrenosum [47]. Another Chinese retrospective analysis of 21 patients with HS recruited from a single Chinese center observed that 76.2% of patients achieved HiSCR50 at week 16 and 71.3% showed a significant reduction in IHS4 compared to baseline. These results improved through week 48, while no serious AEs occurred [48]. Abu Rached et al. reported that 41.9% of the 42 patients after 16 weeks of treatment with secukinumab achieved this outcome [49]. A meta-analysis by Haselgruber et al. on real-world evidence on anti-IL-17 agents approved for HS showed that in 11 studies involving 292 patients treated with secukinumab, 53.3% achieved HiSCR50 at the longest available follow-up, while AEs were reported in 8.2% of cases, with mild pain at the injection site and upper respiratory tract infections being the most common [50].

## 6. Bimekizumab Trials

More recently, bimekizumab, an anti-IL-17A and IL-17F, has been evaluated in several trials for use against HS. A double-blind phase 2 trial was completed by 73 patients with moderate-to-severe disease randomized to receive bimekizumab, placebo, or adalimumab in a 2:1:1 ratio. At week 12, HiSCR50 was achieved by 57.3% of the bimekizumab group, compared with 26.1% of the placebo group. The superiority of the former was also evident in other clinical outcomes such as IHS4, HiSCR75 and HiSCR90 [51]. Only one patient receiving bimekizumab had to discontinue treatment due to ineffectiveness. With regard to safety, 4% of patients receiving bimekizumab developed serious AEs, compared with 10% in the placebo group and 5% in the adalimumab group [51]. BE HEARD I and BE HEARD II are two randomized, double-blind phase 3 studies that evaluated the efficacy and safety of bimekizumab against HS for 48 weeks. A total of 505 and 509 patients aged over 18 years were recruited and randomized 2:2:2:1 stratified according to the worst Hurley stage to receive bimekizumab 320 mg every 2 weeks; every 2 weeks until week 16, then every 4 weeks until week 48; every 4 weeks until week 48; placebo until week 16, then bimekizumab every 2 weeks [52]. The drug proved superior to placebo in both trials. In the first study at week 16, HiSCR50 was achieved by 48% of the bimekizumab group every 2 weeks and by 29% of the placebo group (OR 2.23), while in the second study, it was achieved by 52% and 32% respectively (OR 2.42). In BE HEARD II, considering bimekizumab taken every 4 weeks, the respective values were 54% and 32% (OR 2.42). Responses were preserved at week 48 [52]. Furthermore, in BE HEARD I, HiSCR75 and 90 were achieved at week 16 by 44% and 22% of those receiving bimekizumab every 2 weeks, compared with 18.3% and 11.3% in the placebo group. In the BE HEARD II study, HiSCR75 and 90 were achieved at week 16 in the bimekizumab every 2 weeks group by 38.8% and 19.9% of patients, compared with 15.7% and 5.8% of patients in the placebo group [52]. No new safety signals emerged from the two trials. The most common AEs were HS in both trials, coronavirus infection and diarrhea in the first trial, and headache and oral candidiasis in the second trial. Between the two trials, one death from congestive heart failure was observed in a patient with a cardiovascular history, and bimekizumab was not considered to be related [52]. Several pooled analyses of these trials have integrated various features of HS treatment with bimekizumab. Ingram et al. evaluated the effectiveness of this treatment in relation to the psychological and physical impact that HS has on these patients. They used the HS Symptom Daily Diary (HSSDD) and HS Symptom Questionnaire (HSSQ). Both tools were sensitive to clinical improvements and worsening and correlated strongly with HS disease severity, pain, and quality-of-life measures. They support meaningful interpretation of treatment benefit from the patient perspective [53]. A pooled analysis of the BE HEARD I&II studies on the same endpoints showed a reduction at week 16 in worst HSSDD of −1.9 ± 0.1 in bimekizumab Q2W and −1.9 ± 0.1 in bimekizumab Q4W versus −0.7 ± 0.2 in the placebo group; average skin pain scores were reduced in the same groups by −1.8 ± 0.1, 1.4 ± 0.2, and −0.8 ± 0.2, respectively [54]. Kirby et al. conducted a psychometric evaluation of the Hidradenitis Suppurativa Quality of Life (HiSQOL©) with patients from the two trials. It demonstrates strong psychometric validity, reliability, and responsiveness and proposes as a useful tool in HS clinical trials and potentially in practice for assessing HS-specific quality of life changes [55]. In summary, patients in the trials who received bimekizumab experienced a significantly greater improvement in quality of life compared to the placebo group. The mean HiSQOL score decreased from −11.7 to −10.3 in the bimekizumab group compared to 5.5 in the placebo side [56]. The DLQI minimal clinically important difference (4-point decrease) was also greater among those receiving bimekizumab than in the placebo group (54.9–64.6% vs. 49.1%) [56]. These results were comparable to those of the pain study in these patients, which showed a mean reduction from baseline to week 16 in average skin pain scores of −1.8 ± 0.1 between bimekizumab every 2 weeks and −1.4 ± 0.2 with the drug every 4 weeks, compared to −0.8 ± 0.2 in the placebo group. The scores continued to improve until week 48 [54]. In the open-label extension of the two studies, 446 patients received bimekizumab for up to 2 years. HiSCR50/75/90/100 was achieved by 85.4%/77.1%/57.6%/44.2% of patients, respectively. The most common AEs were consistent with those seen in the one-year studies [57].

## 7. Bimekizumab Real Life

Given the recent approval of bimekizumab, real-life studies need to be increased. Mansilla-Polo et al. observed a significant reduction in certain scores in patients treated with bimekizumab. The HS-PGA fell from 5.1 to 3.2, the IHS4 from 27.1 to 15.6, the DLQI from 21.6 to 12.6, and the VAS scale from 8.3 to 4.7 [58]. A multicenter study of 78 patients treated with bimekizumab evaluated various scores at weeks 16, 24, and 48. IHS4-55 was achieved in 54.7%, 67.9%, and 82.1% of patients at weeks 16, 24, and 48, respectively; IHS4-75 by 41.5%, 30.7%, and 62.5%, respectively; while IHS4-90 by 3.8%, 2.5%, and 32.1% [59]. AEs developed in 34 out of 78 patients (43.6%). The most frequent were mild-to-moderate fungal infections (26.9%) and eczematous reactions (9%), with the eyelid being the most commonly affected site [59]. 10.3% of patients discontinued treatment, mostly due to loss of efficacy (6.4%). Other causes were paradoxical psoriasis, onset of Crohn’s disease, and paradoxical psoriasis (1.3%) [59]. The meta-analysis by Haselburger et al. previously mentioned, for bimekizumab included two real-world studies involving a total of 55 patients. With regard to efficacy, one study reported achievement of IHS-4-55 in 50% of cases at week 16; the other study observed significant reductions in IHS4, DLQI, HS-PGA, and VAS. With regard to safety, AEs were observed in 5.4% of patients, all of which were oral candidiasis [50]. Another retrospective multicenter study of 84 patients treated with bimekizumab observed HiSCR50 achievement in 56% of cases at week 16, with an improvement in DLQI score of 10.7 points and pain scores of 3.4 points. During the same period, IHS-4-55 was achieved by 53.6% of patients. AEs were reported in 20.2% of cases at week 16, mostly candidiasis [60].

## 8. Other IL-17 Inhibitors

Ixekizumab, an anti-IL-17A, and brodalumab, an anti-IL-17 receptor, also belong to the class of IL-17 inhibitors. Currently, these drugs are not indicated for the treatment of HS [9]. Nevertheless, some real-life experiences regarding its use against HS have been published. Frew et al. released an open-label cohort study on 10 patients with moderate-to-severe HS. After 12 weeks of therapy with brodalumab, all patients achieved HiSCR50 and 80% achieved a category change in IHS4, without showing any AEs [61]. In addition, 50% of patients also achieved a HiSCR100. These results were maintained through week 24 [62]. Sonographic markers were also evaluated in the same patients. These were compared with clinical response markers, and it was observed that epidermal thickness was significantly reduced only in patients who achieved HiSCR90 and HiSCR100 and in IHS4 responders. Dermal tunnel diameter was significantly reduced only among HiSCR90 and HiSCR100 responders, but not in IHS4 responders. Finally, Power Doppler intensity, which is correlated with pain and itching, was reduced in all responders when measured with HiSCR50, but not with IHS4 [63]. Serum and skin levels of certain molecules involved in HS inflammatory pathways were also evaluated in these patients. A reduction was observed after 12 weeks of therapy in serum IL-17A and neutrophil-associated lipocalin-2 (LCN2) in the skin [64]. Kearney et al. recruited eight patients who had previously failed at least one biologic. Four of eight biologic-refractory HS patients remained on brodalumab with sustained subjective efficacy and a reduction in mean DLQI from 20.6 to 16.8 at week 16 [65]. Osorio-Gomez et al. conducted a multicentre study recruiting 16 patients with moderate-to-severe HS. After 16 weeks of therapy with brodalumab, 50% of patients achieved HiSCR50 and the mean IHS4 score decreased from 24.13 to 16.81, with no remarkable safety concerns [66]. Some case reports have described the use of brodalumab against HS. Vagnozzi et al. reported the case of a 50-year-old patient who had already failed three other anti-TNF drugs. Brodalumab was therefore started in combination with acitretin, and HiSCR50 was achieved at week 12. At week 48, an IHS4 of 10 was observed, down from 56 at baseline, and at week 136, remission of symptoms was achieved [67]. Yoshida et al., on the other hand, reported the use of brodalumab in a 47-year-old patient with psoriasis concomitant with HS. After 8 months of therapy, they observed a reduction in the Modified Sartorius Score, which evaluates the number, type, and distribution of lesions across affected body areas, from 102 to 30, and a reduction in PASI to zero [68]. Arenbergerova et al. reported the case of a 45-year-old patient with 30 years of HS who had failed several systemic therapies, including an anti-TNF agent. After 3 months of brodalumab, the number of lesions decreased from 27 to 9, the IHS4 score decreased from 62 to 18, and the VAS score decreased from 8.7 to 3.5 [69]. Navrazhina et al. studied HS inflammatory pathway levels in both the skin and serum of 10 patients treated with brodalumab. The authors observed a reduction in inflammatory infiltrate in both lesions and perilesional areas at weeks 4 and 12. During the same period, granulocyte-related inflammatory pathways were also reduced [64].

Regarding ixekizumab, Esme et al. treated five patients with Hurley grade 3 HS who had failed adalimumab. At week 12, 80% of patients (4/5) achieved HiSCR50 and improved DLQI and VAS scores, and no AEs were reported [70]. Gargiulo et al., through a multicentre study of 41 patients, evaluated the efficacy of various anti-IL-17/23 agents against HS. Among these, 5 patients were treated with ixekizumab. HiSCR50 was achieved by 39% of all patients at week 16, 74.3% at week 32, and 77.8% at week 52. However, no data on this outcome are available for patients treated with ixekizumab [71]. With regard to case reports, Megna et al. described the case of a 50-year-old man with HS and psoriasis who had previously failed treatment with methotrexate and unspecified antibiotics. After 10 weeks of brodalumab, the patient achieved HiSCR50, which was maintained until week 24, and PASI also decreased without flare-ups during the same period [72]. Interestingly, Lanzetti et al. reported the case of a 41-year-old woman who developed a streptococcal pharyngeal infection during successful treatment with ixekizumab for HS and psoriasis. Consequently, skin manifestations compatible with Sweet’s syndrome (SS) appeared. The condition was resolved with antibiotic and systemic corticosteroid therapy. The authors reflect that although IL-17 inhibitors are effective in various neutrophilic dermatoses, they were not effective in this case in preventing and counteracting SS [73].

## 9. Discussion

The expanding knowledge of hidradenitis suppurativa (HS) pathogenesis has progressively shifted the therapeutic paradigm from nonspecific immunosuppression to targeted immunomodulation (Table 1).

Among the inflammatory pathways implicated in HS, the IL-17 axis has emerged as a key driver of disease activity. Experimental and translational studies consistently demonstrate increased expression of IL-17 family cytokines within HS lesions, with IL-17A and IL-17F playing a particularly prominent role in sustaining the chronic inflammatory environment. These cytokines promote keratinocyte activation, neutrophil recruitment, and amplification of downstream pro-inflammatory mediators, thereby contributing to the formation and persistence of nodules, abscesses, and dermal tunnels. In current clinical practice, IL-17 inhibitors are primarily considered in patients with an inadequate response or intolerance to anti-TNF agents, particularly adalimumab, which remains the only widely established first-line biologic therapy. Although IL-17 inhibitors have demonstrated efficacy in clinical trials, direct head-to-head comparisons with anti-TNF agents are lacking, making their precise positioning within the therapeutic landscape still uncertain.

The growing understanding of the IL-17 pathway has provided a strong biological rationale for the development of targeted therapies. Clinical trials evaluating IL-17 inhibitors have demonstrated encouraging efficacy in patients with moderate-to-severe HS, a population historically characterized by limited therapeutic options. Secukinumab, a selective IL-17A inhibitor, has shown significant improvements in clinical response, inflammatory lesion reduction, and pain outcomes in large phase III trials, with sustained responses over longer follow-up periods. Similarly, bimekizumab, which simultaneously neutralizes IL-17A and IL-17F, has demonstrated robust efficacy across multiple clinical endpoints, including HiSCR responses, quality-of-life improvement, and pain reduction. 

Emerging evidence suggests that IL-17F may be quantitatively more abundant than IL-17A in HS lesions, supporting the hypothesis that dual inhibition of these cytokines may provide broader suppression of the Th17-driven inflammatory cascade. This concept may partly explain the promising results observed with bimekizumab and highlights the importance of further clarifying the relative contribution of individual IL-17 family members in HS pathogenesis.

Despite these advances, several challenges remain. Treatment responses are heterogeneous, and a substantial proportion of patients experience only partial or inadequate improvement even with biologic therapy. Moreover, although statistically significant, the absolute difference in HiSCR50 response rates between IL-17 inhibitors and placebo remains modest, generally in the range of 10–15% in phase III trials, raising questions about the clinical relevance of these effects in some patients. This variability likely reflects the multifactorial nature of HS, in which genetic predisposition, environmental factors such as smoking and obesity, and dysregulation of multiple immune pathways interact. In addition, patients enrolled in randomized clinical trials are highly selected and may not fully represent real-world HS populations, which often include individuals with more severe disease, multiple comorbidities, and prior biologic failures. Consistently, real-world studies report a broader range of response rates compared with clinical trials, likely reflecting differences in baseline disease characteristics, prior treatments, and treatment adherence. These findings highlight the need for a more personalized therapeutic approach. Future research should focus on the identification of predictive biomarkers of treatment response, optimization of patient selection, and the development of combination or sequential therapeutic strategies.

### 9.1. Critical Appraisal of Evidence: RCTs vs. Real-World Data

#### 9.1.1. Efficacy

RCTs, including large phase III studies, provide high internal validity and demonstrate statistically significant efficacy of IL-17 inhibitors, with HiSCR50 responses generally ranging from 42% to 54% at week 16. These trials benefit from standardized endpoints, controlled conditions, and predefined inclusion criteria. However, the observed placebo-adjusted differences are relatively modest (approximately 10–15%), raising questions about their clinical relevance in routine practice.

In contrast, real-world studies report a wider range of response rates (approximately 40–75%), in some cases exceeding those observed in RCTs. This variability likely reflects differences in patient populations, including disease severity, prior biologic exposure, and comorbidities. While RWE may better capture treatment effectiveness in clinical practice, heterogeneity in study design and outcome measures limits comparability.

#### 9.1.2. Safety

RCTs consistently report favorable safety profiles for IL-17 inhibitors, with mostly mild adverse events such as headache or infections, and no major unexpected safety signals. Their controlled design allows for systematic adverse event reporting but may underestimate rare or long-term risks due to limited follow-up and strict patient selection.

Real-world data generally confirm the acceptable safety profile but provide additional insights into less common or delayed adverse events, such as paradoxical reactions or disease-specific complications (e.g., inflammatory bowel disease). However, underreporting and lack of standardized pharmacovigilance may affect data reliability.

#### 9.1.3. Sources of Bias

RCTs minimize bias through randomization, blinding, and controlled conditions, but they are subject to selection bias, as enrolled patients often represent a highly selected population that may not reflect routine clinical practice. This limits external validity.

Conversely, real-world studies offer greater generalizability but are inherently prone to confounding, selection bias, and information bias, given their observational and often retrospective nature. Differences in baseline characteristics, treatment adherence, and outcome assessment further complicate interpretation.

#### 9.1.4. Overall Interpretation

Taken together, RCTs and RWE should be considered complementary rather than conflicting sources of evidence. RCTs establish efficacy under ideal conditions, while real-world studies provide insight into effectiveness in broader and more heterogeneous populations. The discrepancies observed between these settings highlight the need for cautious interpretation of clinical trial data and reinforce the importance of integrating multiple evidence sources to guide therapeutic decision-making in HS.

#### 9.1.5. Limitations of Available Evidence

Despite the growing body of literature on IL-17 inhibitors in hidradenitis suppurativa (HS), several limitations should be acknowledged. The available evidence is characterized by substantial heterogeneity in study design, patient populations, prior treatment exposure, and outcome measures, which limits direct comparability across studies and precludes quantitative synthesis.

Randomized controlled trials, although methodologically rigorous, include highly selected patient populations and may not fully reflect real-world clinical practice, potentially leading to an overestimation of treatment efficacy. Conversely, real-world studies provide valuable insights into routine care but are often retrospective, involve small sample sizes, and lack control groups, making them more susceptible to bias and confounding.

In addition, follow-up duration is still relatively limited for several agents, particularly newer therapies such as bimekizumab, and long-term effectiveness and safety remain incompletely defined. The absence of validated predictive biomarkers further contributes to variability in treatment response and limits the possibility of a personalized therapeutic approach.

Finally, the potential for publication bias should be considered, as studies with positive findings are more likely to be reported. Overall, these limitations highlight the need for larger, prospective studies with standardized outcomes and longer follow-up to better clarify the role of IL-17 inhibitors in HS management.

## 10. Conclusions

In conclusion, accumulating evidence underscores the central role of the IL-17 pathway in the immunopathogenesis of hidradenitis suppurativa and supports the use of IL-17–targeted therapies as an important treatment option for patients with moderate-to-severe disease [74,75,76,77,78,79,80,81,82]. While agents such as secukinumab and bimekizumab have expanded the therapeutic armamentarium and demonstrated promising clinical outcomes, further studies are needed to clarify their long-term effectiveness, optimize treatment algorithms, and better define their role within the evolving HS management landscape. Overall, although IL-17 inhibitors represent a significant therapeutic advancement, their modest placebo-adjusted efficacy, together with marked interpatient variability and discrepancies between clinical trial and real-world populations, suggests that their overall clinical impact may be more limited than suggested by trial results alone.

## Figures and Tables

**Table 1:** Comparative efficacy and safety of IL-17 inhibitors in hidradenitis suppurativa.

Drug	Target	Study Type	Population	Key Efficacy Outcomes	Safety Profile	Key Notes
Secukinumab	IL-17A	Phase III (SUNSHINE, SUNRISE)	Moderate–severe HS	HiSCR50: ~42–46% at week 16; up to 65% at week 52	Favorable; headache most common AE	Comparable efficacy Q2W vs. Q4W; sustained response
Secukinumab	IL-17A	Pilot/small trials	Severe HS	HiSCR50 up to 70–78% at week 24	No serious AEs	Early efficacy evidence
Secukinumab	IL-17A	Real-world studies	Bio-naïve & experienced	HiSCR50 ~41–75%; meta-analysis ~53%	AEs ~8.2% (mostly mild)	Better in females, lower disease burden
Bimekizumab	IL-17A + IL-17F	Phase II	Moderate–severe HS	HiSCR50 57.3% vs. 26.1% placebo (week 12)	Low serious AEs	Dual inhibition advantage
Bimekizumab	IL-17A + IL-17F	Phase III (BE HEARD I & II)	Moderate–severe HS	HiSCR50 48–54% vs. 29–32% placebo; HiSCR75 up to 44%	Candidiasis, headache; no new signals	Strong multi-endpoint efficacy
Bimekizumab	IL-17A + IL-17F	Open-label extension	Moderate–severe HS	HiSCR50 up to 85.4%	Consistent with trials	Sustained long-term response
Bimekizumab	IL-17A + IL-17F	Real-world studies	Severe HS	IHS4-55 up to 82%; HiSCR50 ~56%	AEs up to 43% (mostly fungal)	Growing real-life evidence

## Data Availability

The data that support the findings of this study are available on request from the corresponding author.
